# Measuring parents' experiences and satisfaction with care during very preterm birth: a questionnaire development study

**DOI:** 10.1111/1471-0528.12925

**Published:** 2014-06-23

**Authors:** A Sawyer, H Rabe, J Abbott, G Gyte, L Duley, S Ayers

**Affiliations:** a Centre for Maternal and Child Health Research, School of Health Sciences, City University LondonLondon, UK; b Academic Department of Paediatrics, Brighton and Sussex University Hospitals TrustBrighton, UK; c Bliss (The Special Care Baby Charity)London, UK; d National Childbirth TrustLondon, UK; e Nottingham Clinical Trials Unit, University of NottinghamNottingham, UK

**Keywords:** Care, experience, factor analysis, preterm birth, questionnaire, satisfaction

## Abstract

**Objective:**

To develop a questionnaire to assess parents' experiences and satisfaction with care during very preterm birth.

**Design:**

Questionnaire development.

**Setting:**

Parents whose babies had been cared for at five tertiary neonatal units in England.

**Population:**

A total of 145 women who gave birth before 32 weeks of gestation, and 85 of their partners.

**Methods:**

A 30-item questionnaire was developed on the basis of qualitative interviews with parents of very preterm babies, a literature review and discussion with relevant experts. The questionnaire was posted to a second group of parents, and its reliability and validity were explored.

**Main outcome measures:**

The Preterm Birth Experience and Satisfaction Scale (P-BESS) was correlated with two global questions measuring satisfaction with care during the birth. Internal consistency was measured using Cronbach's *α*.

**Results:**

Parents of 458 babies were invited to take part and 147 (32%) responded. Two women and 22 partners were excluded or ineligible, leaving 145 women and 85 partners. Factor analysis produced three clear dimensions: Staff professionalism and empathy, Information and explanations, and Confidence in staff. The total scale and three subscales showed high reliability. Strong positive correlations were found between the questionnaire scales and the two global questions, indicating convergent validity. For women whose partners were present at the birth, a fourth factor was identified ‘Partner Involvement’.

**Conclusions:**

The P-BESS appears to be a valid measure of satisfaction with care during very preterm birth.

## Introduction

Preterm birth is the largest risk factor of perinatal mortality and morbidity and is associated with a reduced quality of life, negative psychosocial and emotional impact on the family, and high costs for health services. The highest mortality and morbidity affects the very preterm babies, born at <32 weeks of gestation.^1^ Approximately 1.4% of UK babies are born very preterm, but they account for 51% of infant deaths.^2^ The birth of a very preterm baby can be an extremely stressful and traumatic time for parents.^3^–^6^ The birth is often unexpected and can happen rapidly, and the baby is usually separated from the mother immediately after birth. Understandably these factors have important implications for the parents and healthcare services.

Understanding of the patients' experiences of healthcare services has improved considerably over recent decades, and patient satisfaction is now one of the most frequently reported health outcomes.^7^,^8^ Enhanced satisfaction has been identified as a goal for improvement in health care by the UK government.^9^,^10^ Questionnaires are the most common method of assessing satisfaction. These provide an efficient and cost-effective method of obtaining an overview of patients' experience and allow comparisons to be made between patients and institutions.^11^

Satisfaction with maternity services, especially care during labour and birth, have become increasingly important to healthcare providers, administrators and policy makers.^12^ A number of instruments have been developed to assess women's satisfaction with intrapartum care and childbirth. These include the Labour and Delivery Satisfaction Index,^13^ Perceptions of Care Adjectives Checklist,^14^ and the Intrapartal-Specific QPP-Questionnaire.^15^ However, none evaluate satisfaction with the care for parents of sick or preterm babies.^16^ Giving birth to a very preterm baby is likely to be a different experience to giving birth to a healthy, term baby. For example, a qualitative study exploring parents' views of care during very preterm birth found that staff appearing calm was an important factor of satisfaction, a domain that is not included in current measures of birth satisfaction.^17^ Therefore, current measures of satisfaction with care may not be suitable for such parents.

The aim of this study was to develop a multidimensional questionnaire to assess parents' satisfaction with care during the birth of their very preterm baby.

## Methods

### Preterm birth experience and satisfaction scale (P-BESS)

The objective of this study was to develop a questionnaire to assess parents' experiences and satisfaction with care during the birth of their very preterm baby. The initial questionnaire was developed on the basis of interviews carried out with 39 parents of very preterm babies,^17^ a review of the literature for relevant questions/domains^16^ and a discussion with relevant experts (psychologists, an obstetrician, a neonatologist and user-group representatives). Seven areas of satisfaction with care during preterm birth were identified in the interviews: (i) Information and explanations, (ii) Emotional support, (iii) Encouragement and reassurance, (iv) Staff being confident and in control, (v) Staff being calm in a crisis, (vi) Involvement of the partner and (vii) Birth environment.

An initial collection of 97 potential questions was generated to cover the above seven areas identified in the interviews. This collection included newly constructed questions, and questions from existing birth satisfaction questionnaires for term births. To minimise any response bias, questions were positively and negatively phrased. All of these questions were then screened by two expert reviewers; 30 were chosen, which best covered each of the seven domains identified above and avoided repetition, unclear wording, ambiguous meaning, or overlap with other constructs. Of these final 30 questions: one was from the Labour and Delivery Satisfaction Index;^13^ one was adapted from the Intrapartal-Specific QPP-Questionnaire;^15^ and 28 were constructed specifically for this study. Responses were scored on a five-point Likert scale ranging from 1 (strongly disagree) to 5 (strongly agree). A higher score indicates more satisfaction with the care during the birth. To check face validity, content validity and ease of comprehension the P-BESS was sent to nine parent representatives at Bliss (a charity for premature and sick babies) and a local hospital. As a result of this, minor changes to the wording were made. The reading level of the scale was established as fairly easy to read (Flesch Reading Ease score 79.1).

### Overall satisfaction

To examine the relationship between the P-BESS and overall satisfaction, a series of questions were included. These comprised two rating scales for overall satisfaction with care (‘I was very satisfied with the care during the birth’; ‘The care during the birth could have been improved’) scored on a five-point Likert scale; and three open-ended questions exploring parents' experiences and satisfaction with care (‘Please describe anything about the care during the birth of your baby that you were particularly satisfied with’; ‘Please describe anything about the care during the birth of your baby that you were particularly dissatisfied with’; ‘Is there anything you think the staff could have done differently during the birth of your baby?’).

### Demographic and obstetric information

Questions were also included covering basic demographic (age, ethnicity, education, marital status, employment details), obstetric and neonatal details (parity, previous premature birth or stillbirth, gestation at birth, major complications during pregnancy or labour, type of birth, time since birth, neonatal complications, length of stay in the Neonatal Intensive Care Unit).

### Procedure

Questionnaires were posted to parents with very preterm babies delivered at five tertiary care centres in England. Ethics approval was from the South East Coast—Brighton and Sussex NHS Research Ethics Committee. Parents were eligible for the study if they had a baby born before 32 weeks of gestation and were over 16 years of age. Parents were also eligible if only one member of the couple wanted to take part or if they were single.

Questionnaire packs were sent to parents of babies born in the previous 12 months by the neonatal consultant at each hospital. Information about the study was also available on the neonatal units in the form of posters (although no parents were recruited from the posters). If parents did not respond they were sent a reminder letter and another copy of the questionnaire pack 2–4 weeks later. Bereaved parents were not sent a reminder letter. Parents were also given the option of completing and submitting the questionnaire online (*n *= 19).

### Data analysis

A factor analysis (principal components analysis with direct oblimin rotation) was conducted with the women's P-BESS questionnaires to explore whether questions could be combined into subscales that represent different aspects of satisfaction with care during very preterm birth. Three of the 30 questions asked about partner's involvement in the birth so were only relevant to the women whose partners attended the birth. These questions were therefore excluded from the initial analysis, and 27 questions were entered into the factor analysis. The number of factors to be retained was determined using the scree plot and eigenvalues >1. Questions that loaded on a factor at >0.4 were considered significant and were retained. Questions that loaded on more than one factor ≥0.3 were removed and the analysis was re-run.

To check whether questions and subscales in the women's P-BESS were applicable to partners, a confirmatory factor analysis was conducted. The fit of the women's questionnaire to the sample of partners was evaluated using the following model fit indices: chi-square test, the comparative fit index, and the root mean-square-error of approximation.

There was a minimal amount of missing data (<5%) and missing points were imputed. Analyses were conducted with spss 20 (SPSS Inc., Chicago, IL, USA) and amos 20 (SPSS Inc).

### Validity and reliability

Content validity describes whether an instrument adequately covers the domains to be evaluated. This should be evident through the systematic series of steps taken when designing the P-BESS. Convergent validity refers to the degree to which scores on an instrument correlate with scores on other instruments that measure a similar construct and was explored by examining the relationship between the total P-BESS score (and associated subscales) with two questions assessing overall satisfaction with care during the birth. Reliability of the P-BESS was explored by looking at three indicators of internal consistency: (i) Cronbach's *α* coefficient, which is a measure of the interrelatedness between a set of questions designed to measure an overall construct (a minimum value of 0.7 is considered as acceptable for a new scale);^18^ (ii) corrected item total correlations—this is a correlation of individual questions with the scale total, omitting that question—a coefficient of around 0.3 is considered acceptable;^19^ (iii) the alpha values when individual questions are removed.

## Results

Between May 2012 and August 2012 458 couples/single parents were invited to take part in the study and 147 (32%) returned a completed questionnaire (147 women and 107 partners). Of these, 24 had to be excluded for various reasons: one woman and her partner whose baby was born in an ambulance, another woman who did not complete the questionnaire, and a further 21 partners who were not present at the birth and therefore could not complete the care questionnaire. The final sample therefore consisted of 145 women and 85 partners. Mean time between the birth and questionnaire completion for women was 264 days (SD 126) and for partners it was 266 days (SD 127). For six couples and one woman their baby died after birth. Demographic and obstetric characteristics are shown in Table1.

**Table 1:** Demographic and obstetric characteristics of the women and their partners

	Women (*n *= 145)	Partners (*n *= 85)
**Parent details**		
Ethnicity*		
White European	104 (75)	59 (71)
African	10 (7)	7 (8)
Asian	18 (13)	11 (13)
Other	7 (5)	6 (7)
Marital status**		
Married/Living with partner	115 (83)	78 (95)
Partner but not cohabiting	2 (1)	0 (0)
Separated/Divorced	3 (2)	1 (1)
Single	19 (14)	3 (4)
Education***		
None	7 (5)	9 (11)
GCSEs/O Levels	30 (22)	13 (16)
A-Levels/Diploma/City & Guilds	42 (30)	29 (35)
Undergraduate	26 (19)	15 (18)
Postgraduate	22 (16)	12 (15)
Professional	11 (8)	4 (5)
Employed****	69 (50)	68 (84)
Mean age (SD and Range)*****	31.1 (9.1; 19–44)	24.7 (7.6; 17–60)
**Birth details**		
Pregnancy complications******	79 (56)	
Labour complications*******	28(22)	
Type of birth********		
Emergency caesarean section	62 (43)	
Elective caesarean section	13 (9)	
Vaginal	69 (48)	
Multiple birth*********	23 (16)	
Parity**********		
1	82 (58)	
2	36 (25)	
3	13 (9)	
4+	11 (8)	
Mean gestation (SD and Range)***********	29.3 weeks (2.7; 23–32)	

Number (%) in each group, unless otherwise indicated.

* *n *= 139 for women and *n *= 83 for partners.

** *n *= 139 for women and *n *= 82 for partners.

*** *n *= 138 for women and *n *= 83 for partners.

**** *n *= 137 for women and *n *= 81 for partners.

***** *n *= 138 for women and *n *= 83 for partners.

****** *n *= 142.

******* *n *= 130.

******** *n *= 144.

********* *n *= 143.

********** *n *= 142.

*********** *n *= 142.

### Data screening of questionnaire items

Initial data screening was conducted to remove questions that were not performing well. First, the range of each question was examined using the entire sample (i.e. both parents) and questions were removed that did not use the full range of the scale. This resulted in three questions being removed (‘I trusted the staff to know what was best’, ‘The staff were caring and sensitive’, ‘The staff seemed confident in what they were doing’). Second, the distributions of the questions were also examined through inspection of skewness values and histograms. Satisfaction scales are frequently skewed and this was expected in this sample of parents. All of the P-BESS questions were positively skewed, therefore it was not appropriate to remove any questions on this basis. Finally, questions were screened and removed either if they were too highly correlated with other questions >0.9 (zero questions) or did not significantly correlate with other questions (three questions). These latter questions correlated ≤0.3 with over 80% of questions (‘There were occasions when I was given too much information’; ‘The room felt scary’, and ‘The room was nice’).

### Factor analysis with data from women

The remaining 21 questions were entered into the factor analysis. Statistical checks confirmed the sample was adequate for factor analysis (Kaiser–Meyer Olkin measure = 0.9) and correlations between questions were suitably large [Bartlett's test of sphericity, *χ*^2^ (210) = 2168.1, *P < *0.001]. A further four questions had to be removed because of cross-loadings (‘I felt that the staff were in control’; ‘The staff seemed calm throughout’), low-loadings (‘The staff did not panic’) or because it was the only question loading on a factor (‘Each member of staff introduced themselves’).

The final factor analysis identified three factors with 17 questions as shown in Table2. The three subscales are ‘Staff Professionalism and Empathy’ (seven questions, mean 29.2, SD = 5.1), ‘Information and Explanations’ (seven questions, mean 27.9, SD = 5.7), and ‘Confidence in Staff’ (three questions, mean 12.4, SD = 2.5). The mean score for the total scale was 69.5 (SD = 11.6), out of a possible range of 17–85.

**Table 2:** Factors structure and component loadings of ‘care during childbirth’ questions (*n *= 145)

Questions	Component Loadings		
	Factor 1	Factor 2	Factor 3
**1. Staff professionalism and empathy**			
The staff put me at ease	0.84		
The staff made me feel cared for as an individual	0.82		
There was a pleasant atmosphere in the room	0.82		
The staff were reassuring	0.80		
The staff took control of the situation	0.75		
The staff were encouraging	0.75		
The staff were warm and friendly	0.56		
**2. Information and explanations**			
I was given all the information I needed		0.87	
The staff explained to me what would happen to my baby when he/she was born		0.80	
There were occasions when no one explained to me what was going on		0.77	
The staff explained to me what would happen during the birth		0.72	
The staff kept me informed of what was happening		0.69	
I understood what was happening		0.60	
The staff explained everything really well		0.56	
**3. Confidence in staff**			
I did not have confidence in the staff			0.82
The staff did not understand how I was feeling			0.69
The staff did not listen to what I had to say			0.68
**Eigenvalues**	8.7	1.6	1.2
**% Variance explained**	51.4	9.6	7.0
4. Partner involvement*	**Factor 4**		
The staff encouraged my partner's involvement	0.79		
The staff involved my partner in what was going on	0.74		
My partner felt in the way throughout**	0.61		

Instructions were provided as follows: This questionnaire asks you about your experiences and satisfaction with care at the *birth* of your premature baby. Please read each statement carefully and indicate the extent to which you agree or disagree with each question. If you had a caesarean section under general anaesthetic then we understand that some of these questions may be difficult to answer but please complete as best you can.

* This analysis was performed only with the women who were able to complete the partner involvement questions (*n *= 108).

** Removal of this item increased scale reliability from 0.72 to 0.91, therefore we recommend this item is removed from this subscale.

### Inclusion of partner involvement subscale

The factor analysis was re-run (*n *= 108) with the addition of the three partner involvement questions (and the 17 questions described above). This confirmed that the same three factors reported above remained, with the addition of a fourth factor that included the questions regarding involvement of partner (three questions). This additional factor is reported at the end of Table2 and is labelled ‘Partner Involvement’.

### Reliability of subscales

The total scale and subscales had good reliability with all Cronbach's *α* above the acceptable level of 0.7 (*α* 0.94 for the total scale, 0.92 for Staff Professionalism and Empathy, 0.89 for Information and Explanations, and 0.77 for Confidence in Staff). All item-total correlations were above 0.3, indicating that individual items correlate well with the total scale. Reliability for the Partner Involvement subscale was 0.72 but deletion of the question ‘My partner felt in the way throughout’ increased reliability to 0.91. This question was therefore removed from this subscale.

### Validation of questionnaire with women

Convergent validity was explored by examining the relationship between the P-BESS scale and the questions measuring overall satisfaction with care and the need for improvement. Total scores on the P-BESS were related to higher levels of overall satisfaction (*r*_*s*_ = 0.73, *P *< 0.001) and less need for improvements (*r*_*s*_ = −0.56, *P *< 0.001). Staff Professionalism and Empathy was related to higher levels of overall satisfaction (*r*_*s*_* *= 0.63, *P* < 001) and less need for improvements (*r*_*s*_ = −0.43, *P *< 0.001). Information and Explanations was related to higher levels of overall satisfaction (*r*_*s*_ = 0.69, *P *< 0.001) and less need for improvements (*r*_*s*_ = −0.52, *P *< 0.001). Confidence in Staff was related to higher levels of overall satisfaction (*r*_*s*_ = 0.55, *P* < 0.001) and less need for improvements (*r*_*s*_ = −0.59, *P *< 0.001). Convergent validity was also examined for the Partner Involvement subscale (*n *= 108). Partner Involvement was related to higher levels of overall satisfaction (*r*_*s*_ = 0.60, *P *< 0.001) and less need for improvements (*r*_*s*_ = −0.41, *P *< 0.001).

### Confirmatory factor analysis: partners

To check whether the P-BESS scores and subscales were applicable to partners a confirmatory factor analysis was conducted. Results showed that although the scale was reliable (*α* = 0.93) the three subscales identified in women's responses were not applicable to partners. Fit indices revealed that the three factor solution did not fit the partner's data well (*χ*^2^ = 222.9, *P *< 0.001, root mean-square-error of approximation 0.102, comparative fit index 0.86).

We therefore recommended that only the total score on the satisfaction with care measure is used for partners. The mean score for the total scale was 67.5 (SD = 9.5). Total scores on the questionnaire were related to higher levels of overall satisfaction (*r*_s_ = 0.72, *P *< 0.001) and less need for improvements (*r*_s_ = −0.61, *P* < 0.001) indicating convergent validity in partners.

### Relationship between satisfaction with care and demographic and birth variables

The majority of demographic variables were not associated with total scores on the *P*-BESS, or the subscales. However, women who were not working were generally more satisfied with staff provision of information and explanations (mean = 29.0 SD = 5.0) than women who were working (mean = 26.6, SD = 6.3, *U* = 2.2, *P *< 0.5). There was an effect of birth type on overall satisfaction with care [*H*(4) = 11.6, *P *< 0.05] indicating that women who had an emergency caesarean section were more satisfied with their care overall (mean* *= 73.5, SD = 12.0) than women who had a vaginal birth (mean* *= 66.8, SD = 12.0). Women were also more satisfied with staff professionalism and empathy [*H*(4) = 10.4, *P *< 0.05] and confidence in staff [*H*(4) = 11.4, *P *< 0.05] if they had an emergency caesarean section compared with a vaginal birth. Women who reported complications during pregnancy were more satisfied with their care overall (mean = 71.5, SD = 11.0) in comparison to those who did not report complications (mean* *= 67.2, SD = 12.1), *U* = −2.3, *P *< 0.05. Women were also more satisfied with information and explanation *(U *= −2.0, *P *< 0.05) and confidence in staff (*U *= −2.4, *P *< 0.05) if they reported complications during pregnancy. Time since birth and completion of the questionnaire was not associated with the total score, or any of the three subscales (*P* values >0.05). Women's scores on the P-BESS and individual subscales did not differ significantly across the five centres.

In partners, age was positively associated with overall satisfaction, with older partners reporting higher levels of satisfaction (*r*_s_ = 0.22, *P *< 0.05). Partners' satisfaction with care at birth was not associated with time since birth, gestation, and whether the baby was alive or not. Partners' scores on the P-BESS did not differ significantly across the five centres.

## Discussion

### Main findings

The aim of this study was to develop a questionnaire that can be used to assess parents' experiences and satisfaction with care during the very preterm birth. Currently, there are no measures that have been developed specifically for preterm birth. The P-BESS consists of 17 questions with three clear subscales (Staff Professionalism and Empathy, Information and Explanations, Confidence in Staff). The total scale and all three subscales showed high reliability and there was evidence for validity. A fourth subscale can be added to assess Partner Involvement for women whose partners' attended birth.

The factor analysis provided support of the P-BESS being multidimensional. The three identified domains are consistent with the literature, interviews with parents and input from healthcare professionals. The first subscale *Staff Professionalism and Empathy* explained the largest proportion of variance. This is not surprising as support from staff is widely recognised as an important factor in determining birth satisfaction. Also, the stress and uncertainty surrounding very preterm birth are likely to increase the need for emotional support and reassurance from the staff.^20^ Likewise, the other two subscales, *Information and Explanations* and *Confidence in Staff* have been previously identified as important factors of satisfaction for during very preterm birth.^17^ Correlations between the questionnaire scales and the two global questions of satisfaction provide support for the convergent validity of the questionnaire. Britton^21^ proposes that an ideal measure of perinatal satisfaction is one that includes questions that assess global satisfaction and specific domains of care. Therefore it is recommended that researchers include the two global questions alongside the 17 specific questions. The scale was also reliable, as indicated by Cronbach's *α*, and there were strong correlations between individual questions and scale scores.

### Strengths and weaknesses

To our knowledge the P-BESS is the first questionnaire to assess satisfaction with care during very preterm birth. The P-BESS was comprehensively developed, can be completed quickly, administered by post or in the hospital, and is easy to score. The questions and domains were derived from parent interviews, discussion with healthcare professionals, and pilot tested with mothers. Also, the wording of many of the questions was based on interviews with women.^17^ The qualitative responses provided by the parents did not suggest any additional areas of satisfaction that were not covered by the questionnaire. This all suggests good face and content validity. A further advantage of this questionnaire is that it was also administered to and validated on partners.

Limitations include a relatively low response rate (approximately 30%), although this is a good response for studies of this kind. Also the sample size was relatively small for a factor analysis, which limits the validation process of the questionnaire. Moreover, the sample is not representative of all parents who have had a very preterm birth. For example, the sample mainly consisted of white, highly educated, married/co-habiting women. This is especially relevant because there is evidence to suggest that there is a higher incidence of very preterm birth in certain ethnic groups^22^ and in women from very deprived areas.^23^ Therefore further studies are needed to test the refined instrument in a larger, more representative sample of parents, which includes seldom heard groups, who have given birth to a very preterm baby. Finally, as the same factor structure was not identified in partners as women it is advised that only the total score is used, which means the individual factors of care cannot be explored for partners. However, the total scale does have high reliability and demonstrates convergent validity.

### Interpretation

Consistent with other studies of maternity satisfaction^24^,^25^ parents reported that they were very satisfied with the care. Parents may have felt reluctant to criticise the professionals who had taken care of them and their premature baby. This ‘halo effect’ may be even more evident for parents of very premature babies as the staff have been looking after their baby for many weeks.^24^ It is also possible that parents' experiences of their time on the Neonatal Intensive Care Unit may influence their birth satisfaction ratings.^21^ Similarly, some researchers raise the issue that women do not know what care during birth *should* be like and therefore just evaluate the *status quo*.^25^,^26^ Furthermore, although parents were instructed to return the questionnaires to the researcher (who was not associated with the hospitals), the letter of invitation was sent by a neonatal consultant, which may have influenced ratings. However, there was a wide range of responses, with the lowest score being 29 and the highest 85, which suggests that the measure can discriminate among women with different satisfaction ratings.

Some studies suggest that the timing of administering the questionnaire may have an influence on satisfaction. In the current study parents completed the questionnaire approximately 9 months after the birth. Studies suggest that parents' reports may be less positive 7–12 months after birth, compared with the first 6 months.^25^ Assessment of satisfaction with childbirth may be more suited when a certain time lag has passed following birth as this will give the woman time to reflect on her experience and decide whether she was satisfied.^16^ However, satisfaction assessed early on may be particularly influenced by expectations, and as many very preterm births are unexpected this could have a negative impact on satisfaction ratings.^21^ It should also be noted that the total score and all three domains of satisfaction were not related to the time following birth. Future studies that use the questionnaire should assess the potential impact of timing of administration.

The factor structure identified using the women's data did not fit with the partner's data well. There are a number of possible explanations for this. First, the questions were based primarily on previous interviews with parents^17^ and current literature on maternity satisfaction. In the interview study only seven fathers were interviewed (compared with 32 mothers) and most literature has only focused on mothers' experiences with care.^27^ Studies suggest that fathers' experiences of preterm birth differ from those of mothers,^28^ which could therefore also influence fathers' evaluation of care. Another possible explanation is that the sample size for a confirmatory factor analysis was small.^29^ Fathers of sick, preterm babies are recognised as a difficult group to recruit into research^30^ and increased efforts are needed to ensure that their views are adequately represented.

## Conclusion

In summary, this study reports the development and testing of the first questionnaire to assess satisfaction with care during very preterm birth. The P-BESS has three domains that are consistent with previous research and include important components of satisfaction. Depending on the needs of the researcher/clinician, questions can be summed to produce a total score, or factors can be looked at individually. A total score may be useful to compare across hospitals and differing practices, whereas individual aspects of the care environment can be evaluated using the separate subscales. The findings suggest good reliability and validity. Recommendations for future testing of the P-BESS include testing in a larger and broader population, and further testing of the construct validity.

### The very preterm birth collaborative group

Project Group: Alexandra Sawyer, Heike Rabe, Jane Abbott, Gill Gyte, Lelia Duley, Susan Ayers; Principal Investigators at each site: Narendra Aladangady, Jon Dorling, Joe Fawke, Heike Rabe, Tim Scorrer; Research/Neonatal Nurses: Gillian Ager, Johnette Brown, Valerie Harper, Marie Hubbard, Liz Lance.

### Disclosure of interests

The authors declare that there are no conflicts of interest.

### Contribution to authorship

AS contributed to the protocol, co-ordinated the study, analysed the data and drafted the manuscript; HR contributed to the protocol and the revision and final approval of the manuscript. JA and GG (representatives from parent groups) contributed to the protocol and the revision and final approval of the manuscript. LD designed the study, contributed to the protocol and the revision and final approval of the manuscript and SA designed the study, contributed to the protocol, data analysis, revision and final approval of the manuscript.

### Details of ethics approval

The study received approval from the NRES South East Coast—Brighton and Sussex NHS Research Ethics Committee. Reference: 12/LO/0145. Date of approval: 8 February 2012.

### Funding

National Institute of Health Research (Reference RP-PG-0609-10107): Improving quality of care and outcome at very preterm birth. This paper presents independent research funded by the National Institute for Health Research (NIHR) under its Programme Grants for Applied Research funding scheme (RP-PG-0609-10107). The views expressed in this paper are those of the author(s) and not necessarily those of the NHS, the NIHR or the Department of Health.
